# Identification and characterization of a case of mild familial partial lipodystrophy in a carrier of a LMNA p.Arg582Leu variant

**DOI:** 10.1007/s00592-024-02396-w

**Published:** 2024-10-29

**Authors:** Anna Maria Barile, Cristiana Randazzo, Francesca Di Gaudio, Carola Buscemi, Giuseppina Brunacci, Silvio Buscemi

**Affiliations:** 1 Dipartimento Di Promozione Della Salute, Materno-Infantile, Medicina Interna E Specialistica Di Eccellenza (PROMISE), University of Palermo, Piazza Delle Cliniche, 2-90127 Palermo, Italy; 2 Unit of Clinical Nutrition, Obesity and Metabolic Diseases, AOU Policlinico “P. Giaccone”, Palermo, Italy; 3 Unit of Chromatography and Mass Spectrometry Section, Quality Control and Chemical Risk (CQRC), Villa Sofia-Cervello Hospital, Palermo, Italy

**Keywords:** Lipodystrophy, Dunnigan syndrome, Adiposopathy, Insulin resistance

## Introduction

Laminopathies are a group of rare disorders caused by mutations in the Lamin A/C gene located on human chromosome 1q22. The *LMNA* gene encodes Lamin A and C that are components of the nuclear lamina in most differentiated cells. Furthermore, Lamin A/C plays a pivotal role in cell differentiation. *LMNA* mutations affect mainly mesenchymal tissues (muscle, bone, and adipose tissue), although they sometimes also affect the nervous system to varying degrees. There are several clinical presentations of laminopathies, as different *LMNA* mutations are responsible for different phenotypes, including lipodystrophy, skeletal muscle dystrophy, and cardiomyopathy [1]. *LMNA* lipodystrophy can be classified as partial, or generalized, based on the extent of atrophy of the subcutaneous fat. The most well-known and common form of *LMNA* lipodystrophy is familial partial lipodystrophy type 2 (FPLD2) that is transmitted as an autosomal dominant disorder. FPLD2 is characterized by subcutaneous adipose tissue loss from the trunk, buttocks and limbs and muscular pseudohypertrophy which is usually accompanied by accumulation in the face, neck and abdominal viscera, and is frequently associated with metabolic and cardiovascular complications. The reported prevalence is 1 case per 100,000 inhabitants in Europe, but it is likely largely underestimated. We herein report the case of a female patient who was occasionally found to harbor a mutation compatible with FPLD2.

## Materials and methods

**Fig. 1:**
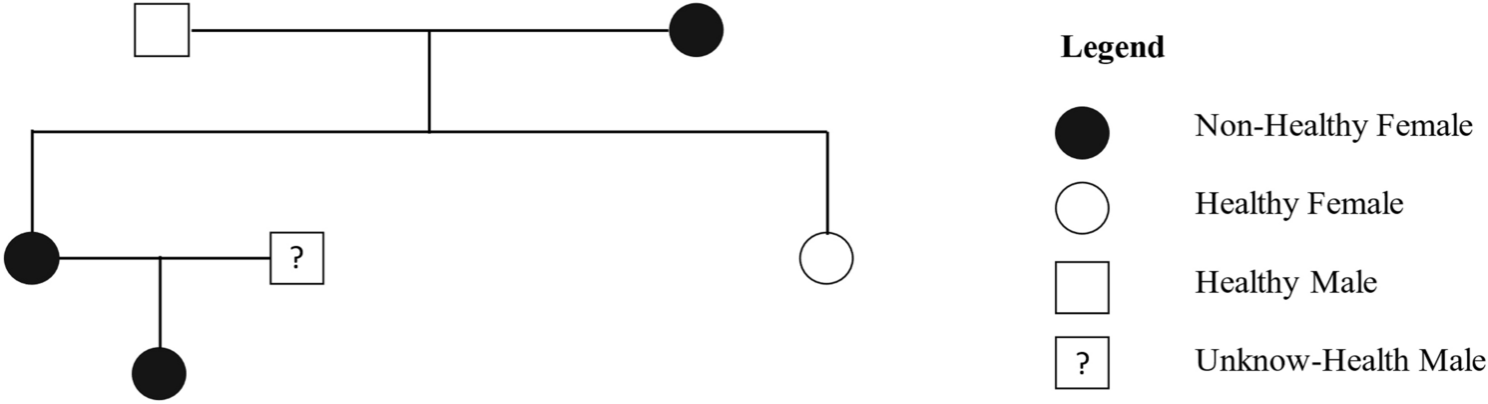
Genealogical tree study of the carrier of the *LMNA* p.Arg582Leu variant

The patient, aged 38 years, was referred to the Unit of Clinical Nutrition, Obesity and Metabolic Diseases of the University Hospital Policlinico “P. Giaccone” in 2022 by the geneticist of the Unit of Chromatography and Mass Spectrometry Section, Quality Control and Chemical Risk (CQRC), Villa Sofia-Cervello Hospital, Palermo, Italy, following a clinical exome procedure that revealed the presence of a mutation in the *LMNA* gene, defined as a missense variant in exon 11 c.1745G>T and consisting of the amino acid change p.Arg582Leu. Additionally, the parents and her 3-year-old daughter underwent genetic investigations (Fig. 1) after providing written informed consent. Our index case had a sister who denied her consent for genetic analysis and was available only for anamnestic information and physical examination.

Different measurements were performed. Briefly, anthropometric measurements included height, body weight, body mass index [BMI; body weight (kg)/height^2^ (m^2^)], body composition in terms of fat mass (FM) and fat-free mass, which were estimated via dual-energy X-ray absorptiometry (DEXA; Hologic Serie Discovery; Bedford, MA, USA); the latter also permitted measurement of segmental (legs, arms, trunk) body composition. Abdominal visceral and subcutaneous adipose sizes were estimated via high-resolution B-mode ultrasound (EPIC 5; Philips; US) as cutis-rectis muscles thickness (CR), a measure of subcutaneous fat, and as rectis muscles-aorta thickness (RA), a measure of visceral abdominal fat. Skinfold thicknesses (subscapular, suprailiac, tricipital, bicipital, midthigh, and calf) were measured (caliper; Holtain Ltd., Ceosswell, UK) as indicators of subcutaneous fat size. Indirect calorimetry was used to measure the resting energy expenditure (REE) and the mixture of carbohydrates, lipids and proteins for basal oxidation (Quark RMR; Cosmed, Rome, Italy); briefly, gas exchange measurements were continuously obtained for at least 1 h in the morning after about 12 h overnight fasting and in stable body weight conditions for at least 1 month. The clinical exome procedure was performed at the Unit of Chromatography and Mass Spectrometry Section, Quality Control and Chemical Risk (CQRC) of the Villa Sofia-Cervello Hospital (Palermo, Italy). All laboratory determinations were carried out after at least 12 h of fasting. Leptin was measured by ELISA from Mediagnost, Reutlingen, Germany. Total adiponectin was measured by ELISA from Merck KGaA, Darmstadt, Germany. Other biochemical values were determined via automated equipment at the Laboratory Medicine Unit at the University Hospital of Palermo, Italy.

## Case presentation

The patient was born by nonconsanguineous parents after a normal pregnancy (birth weight 3.1 kg). She had regular psychomotor development, menarche at 11 years of age and subsequently irregular menstrual cycles with oligomenorrhea. She was diagnosed with polycystic ovary syndrome and periodically took estrogen-progestins both for contraceptive purposes and to regulate her cycles, but she often had to suspend them owing to the appearance of severe hypertriglyceridemia (> 1000 mg/dl in one episode occurring at 25 years of age) and hypertransaminasemia. She had a pregnancy at 35 years of age that was complicated by gestational diabetes and resulted in eutocic delivery of a 2.7 kg baby girl. After pregnancy, her menstrual cycles became regular. She was also referred as a carrier of Gilbert's hyperbilirubinemia, a small cyst in the right lobe of the thyroid, and mild allergic asthma. At the age of 28, she underwent breast augmentation surgery. She had never been a smoker, did not usually consume alcohol and referred to adhering to a healthy lifestyle according to the Mediterranean diet, being physically active (moderate physical activity at least 3 times a week for approximately 1 h). In 2021, she experienced syncope, and Brugada syndrome was suspected; consequently, she underwent genetic testing. Gene mutations associated with the Brugada syndrome were not identified, and until now, no other syncopal episodes have occurred, but a c.1745G>T; (p.Arg582Leu) NM_170707.4 variant of the lamin gene was detected. Indeed, she presented with some traits that were suggestive of partial lipodystrophy, such as an abnormal distribution of subcutaneous fat at physical examination, with scarce accumulation in the abdominal wall, legs, arms and gluteal region, with muscle pseudohypertrophy. The BMI was within the normal range (21.8 kg/m^2^). DEXA revealed a normal value (23.5%) of total fat mass size, and the segmental body fat distribution indicated the same compartment composition in different body segments, ranging from 17.4 to 27.4% (FM: left arm = 20.0%, right arm = 17.4%, left leg = 27.4%, right leg = 27.2%, trunk = 22.3%). Interestingly, skinfold measurements (skinfolds: triceps = 7 mm, biceps = 4 mm; suprailiac = 9 mm, subscapular = 19 mm; midthigh = 5 mm) indicated the paucity of subcutaneous fat; thus, we must assume that most body fat was visceral fat (in the case of truncal fat) or intramuscular fat, suggesting ectopic fat accumulation. Additionally, the echo-derived measures of the thickness of the rectis-aorta (RA = 59 mm) versus the value of the cutis-rectis thickness (CR = 6 mm) were consistent with the presence of a significant fat mass in the abdomen that was located in the visceral compartment. Indeed, ectopic body fat accumulation was also suggested by echo-diagnosed liver steatosis (with a normal stiffness of 2.8 kPa according to shear-wave elastography), despite normal body weight and total fat mass values. The results of laboratory tests related to glucose tolerance and different biochemical and hormonal data are presented in Table 1. In particular, the HOMA-I score was slightly high, and insulin concentrations reached high values in the last hour of the oral glucose tolerance test (OGTT), suggesting insulin resistance; furthermore, the HDL-C levels were below the normal range. Leptin and adiponectin serum concentrations were within the range of normal-weight women. According to indirect calorimetry, the patient had a normal basal energy expenditure (1718 kcal/24 h; 33.7 kcal/kg-fat free mass × 24 h) and exhibited very high basal lipid oxidation with almost negligible carbohydrate oxidation (carbohydrates 0.2%, lipids 78.0%, proteins 21.8%). The echocardiography revealed normal heart size, geometry and function (ejection fraction 60%) with a mild alteration in diastolic transmitral flow (e/e′ = 7). Additionally, cardiac magnetic resonance imaging was performed, and the findings were normal.

**Table 1:** Biochemical and hormonal data

	Patient	Mother	Normal value
HbA_1_c (%)	5.4	5.9	< 5.7
OGTT, glucose (mg/dl)			
Fasting	96	107	< 100
60 min	159	268	
90 min	129	245	
120 min	117	224	< 140
OGTT, insulin (mIU/L)			
Fasting	11.7	42.1	2.6–24.9
60 min	123	432	20–120
90 min	207	613	20–90
120 min	224	705	20–70
HOMA-index	2.77	11.12	0.3–2.5
Serum concentrations of	1.47	0.31	0.27–4.2
TSH (mIU/L)	1.47	0.31	0.27–4.2
FT4 (ng/dL)	1.37	1.19	0.7–1.7
Cholesterol (mg/dl)	179	238	< 200
HDL-cholesterol (mg/dl)	34	48	> 50
LDL-cholesterol (mg/dl)	127	135	< 116
Triglycerides (mg/dl)	88	277	< 150
AST/ALT (U/L)	29/22	37/37	< 35/35
γ-GT (U/L)	10	65	5–36
Vitamin D (ng/ml)	59.9	-	30–100
Leptin (μg/mL)	12.8	3.6	2.43–28.0 (BMI 20–25) 5.07–58.3 (BMI 25–30)
Adiponectin (μg/ml)	6.5	2.2	5–37 (BMI < 25) 2–20 (BMI > 30)

*ALT* alanine aminotransferase, *AST* aspartate aminotransferase, *BMI* body mass index (kg/m^2^), *γ-GT* gamma-glutamyl transferase, *HbA*_*1*_*c* glycated hemoglobin, *HDL* high-density lipoprotein, *HOMA-index* homeostasis model assessment index (fasting plasma glucose x fasting plasma insulin/405), *LDL* low-density lipoprotein, *OGTT* oral glucose tolerance test

Her family members were subsequently investigated, except for her sister, who had no phenotypic signs of lipodystrophy and denied consent for further investigation. The 73-year-old father was affected by type 2 diabetes, hypertriglyceridemia, and hypercholesterolemia. Her mother was 64 years old and was underweight before the first pregnancy. She was also affected by hypertension since she was 44 years old. At 61 years of age, localized scleroderma was diagnosed, and she reported anxiety and depression that periodically required drug treatment, with autoimmune multinodular goiter with hypothyroidism on treatment with l-thyroxine. Physical examination revealed features consistent with partial lipodystrophy. She was overweight (BMI = 28.9 kg/m^2^), exhibited high values of fat mass (DEXA 38.9%) mainly located in the truncal segment (FM: left arm = 39.2%, right arm = 40.1%, left leg = 29.5%, right leg = 29.3%, trunk = 44.7%) and less in the arms and legs (skinfolds: triceps = 11.5 mm, biceps = 7 mm; suprailiac = 36.5 mm, subscapular = 26 mm; midthigh = 5.5 mm). The results of the OGTT and other blood biochemical and hormonal measurements for patient’s mother are reported in Table 1. Severe insulin resistance with type 2 diabetes, hypertriglyceridemia, and hypercholesterolemia were demonstrated. Genetic investigation revealed that the variant c.1745G>T (p.Arg582Leu) identified in the index case was found in her mother and her daughter (Fig. 1), the latter presenting no clinical evidence of the disease.

By using appropriate databases (Varsome and Franklin) this variant can be defined as likely pathogenic or pathogenic, respectively. The gnomAD v4.1.0 database reports an allele frequency of 1 out of 1,179,788 in a European (non-Finnish) population. These data reinforce the conclusion that the p.Arg582Leu *LMNA* variant is indeed likely causative in this family.

## Discussion

Type 2 familial partial lipodystrophy (FPLD2) is a laminopathic lipodystrophy that may recognize different mutations within the *LMNA* gene [2]. The most common form of the disease is also known as the Dunnigan-subtype. Patients carrying different mutations have also been described with varying phenotypes, with or without progeroid features. In the present case report, we identified a missense variant in exon 11, c.1745G>T, consisting of an Arg582Leu mutation, which was previously described in a man with a severe metabolic phenotype associated with visceral fat accumulation but no evidence of subcutaneous lipoatrophy [3]. Furthermore, Besci et al. reported this mutation in a 44-year-old woman with a BMI of 28 kg/m^2^, liver steatosis and leptin levels of 4.6 ng/ml [4]. The case of our patient is characterized by a paucity of subcutaneous adipose tissue accumulation in arms, legs and abdomen (interestingly, she underwent surgery for breast augmentation). The patient was prescribed a low-fat and low-glycemic-index Mediterranean diet. Most likely, as a consequence of the difficulty in storing lipids in subcutaneous adipose tissue and the low-fat content of her diet, basal lipid oxidation was very high, whereas carbohydrate oxidation was low. In fact, very high lipid oxidation may be a compensatory mechanism to reduce lipotoxicity, as previously reported [5]. The serum concentrations of leptin and adiponectin were within the range of normal weight women. Therefore, the amount of adipose tissue that we measured allowed normal leptin production that permitted adequate reproductive function and appetite control, facilitating adherence to the diet. An interesting point is the comparison with patient’s mother. She had a higher BMI and total fat mass but low arm and leg subcutaneous fat, very high insulin resistance with type 2 diabetes and hypertriglyceridemia, and liver steatosis with increased blood concentrations of liver enzymes. Interestingly, she presented low serum concentrations of leptin and adiponectin. These data are indicative of an unfavorable metabolic evolution of the disease with aging. Despite lipodystrophy, the cardiological exams excluded ongoing cardiac complications and, to date, she never exhibited heart disease. Also, we activated a strict follow-up for patient’s daughter for the possible anticipation of the syndrome.

For these patients, new therapeutic options, such as GLP-1 receptor agonists (i.e., semaglutide), would be of interest because of their possible metabolic and cardiovascular beneficial effects, particularly those related to appetite and adiposopathy (in the latter case, beneficial effects have also been attributed to glitazones); however, this possibility is only speculative.

The case of our patient (but also of her mother and possibly of her daughter) was an occasional diagnosis; these cases emphasize the importance of identifying lipodystrophies associated with lamin gene mutations (and possibly other genes), which may be responsible for a higher proportion of patients with metabolic syndrome and type 2 diabetes then generally thought. Describing newly identified cases is useful to improve the knowledge and, possibly, treatment of these conditions.
